# *An Account of a Case of Pulmonary Consumption Successfully Treated by a Salivation; in a Letter from* George Pfeiffer, M. D. *to* Dr. Benjamin Rush

**Published:** 1802-10-01

**Authors:** George Pfeiffer

**Affiliations:** Philadelphia


### *An Account of a Case of Pulmonary Consumption successfully treated by a Salivation; in a Letter from* GEORGE PFEIFFER, M.D. *to* Dr. BENJAMIN RUSH.

DEAR SIR,

ALTHOUGH, in medicine, as in other sciences, the limited faculties, and, consequently, the limited knowledge of man, precludes the probability of his ever being able to form a perfect system, still does it afford matter of pleasure and encouragement to reflect, that by his experience, and the exercise of his reason, aided by those fortunate accidents which have always had so great a share in the prosperous issue of his affairs, he has been progressively conducted to the attainment of grand and important truths—truths by which many of the evils incident to to his condition here have been ameliorated or deftroyed, and new fources of happinefs opened to him.

One of the moft pleafing fubjects of contemplation afforded by the prefent period, fo rich in moral and phyfical improvements, is the profpect of bringing into fubjection to our art, a difeafe which has hitherto baffled the collected experience and fkill of the whole medical world, and annually given to the grave its thoufands and tens of thoufands of victims.

This profpect was prefented to my mind by the perufal of three letters, written by you to Dr. Miller, of New York, on the falutary effects of a falivation, and alfo of tonic remedies in pulmonary confumption.

Do not think I mean to flatter you, Sir, when I declare that I never read any thing in my life which excited a feeling more nearly approaching to ecftacy, than did that little production.

Inftantly I determined to avail myfelf of fo promifing a practice in the very firft confumptive cafe that fhould offer itfelf to my attention.

Befides feveral doubtful and complicated cafes, I have lately met with one which, to me, appears unequivocally to have been a true phthifis pulmonalis.

In the hope that it will give you fome fatisfaction, I take the liberty of tranfcribing the hiftory of it for your perufal.

——— Will, aged fifty-five years, a tall, flender man, by trade a houfe carpenter, when labouring under a catarrh, in the fummer of the year 1794, was fudddenly feized, during a violent fit of coughing, with an alarming hæmoptoe, by which he loft a confiderable quantity of blood, and his ftrength was much reduced. It was foon checked, however; and though there were feveral flight returns of it, he, in a few weeks, recovered his ufual fhare of health, and followed his trade without inconvenience, till the autumn of 1798, when he fuffered an attack of the yellow fever. Since that period he thinks he never has felt fo ftrong as he did before; and through the courfe of the laft fummer his ftrength declined fo rapidly, that by the beginning of September he was fcarcely able to continue his work; and towards the end of the fame month he was feized, as he thought, with the bilious fever, which was then the reigning epidemic in Philadelphia, and very prevalent in the Northern Liberties, where he refides. But the fymptoms of his diforder, from the very beginning of his confinement, fo far as I have been able to collect them, both from his own mouth and his wife, inftead of bearing the character of our autumnal remittent, were rather characteriftic of hectic fever and advanced pulmonary confumption. They were frequent and irregular alternations of chills, fever and fweats, with pain in the thorax, and and a diftreffing heavy cough, which was moft fevere at night, attended with a copious expectoration of pus-like matter. There was alfo a fteady, and often an excruciating pain, in his left hypogaftric region, but no perceptible enlargement. Under thefe fymptoms I found him labouring early in December, when I firft faw him, and fo weak as to be unable to rife from his bed without affiftance.

A few minutes at his bed-fide were fufficient to imprefs my mind with the moft thorough conviction that he was far advanced in phthifis, and that, without fpeedy relief, he would probably not live more than a few weeks, and certainly not till fpring. He had taken fome medicines, amongft others nitrous powders; but without any relief.

I immediately prefcribed your nitrous antimonial powder, with a little opium, and from half a grain to a grain of calomel in each, and directed him to take three powders daily. At night he took an antimonial anodyne draught; and on the painful fide I ordered a quantity of warm fpirits, or camphorated fpirits, to be rubbed feveral times a day. This courfe was purfued for about ten days, when, obferving no effect to be produced on his mouth by the powders, I ordered, in addition to them, ftrong mercurial ointment to be applied to his fide twice a day, inftead of the camphorated fpirits.

After an ounce of the ointment had been ufed, a gentle falivation began, which continued about three weeks, with fcarcely any inconvenience to the man, who took no other medicine, after the falivary difeafe was induced, but a paregoric draught occafionally.

As the fpitting progreffed, all the fymptoms of his diforder gradually fubfided; and, by the time it ceafed, his irregular chills, fever and fweats, as well as the purulent expectoration, and the pain in his fide, had entirely left him. Only a flight cough remained, which was foon vanquifhed by the paregoric draughts above mentioned. His appetite and ftrength returned, as if by a charm, and, on going to his houfe a few days afterwards, I was both aftonifhed and delighted to find him hard at work with his faw. He declared he felt perfectly well, and was not at all fatigued by his labour. I advifed him to take two or three glaffes of Madeira wine every day, for fome time, to keep his bowels open, and wear a flannel fhirt next to his fkin. I faw him, for the laft time, on the 23d inftant, when he ftill continued in perfect health.

Thus, Sir, have I given you a faithful account of what, to me, appeared a defperate cafe of difeafe, and which I had very faint hopes of being able to conquer, even with the aid of the Hercules of Medicine, although I had your powerful teftimony in its favour, and my own knowledge of its general and unexampled efficacy.

As

As the iſſue has turned out, I contemplate myſelf as having been, through you, the humble inſtrument of reſcuing a fellow creature from certain death.

Believe me, dear Sir, I conſider myſelf greatly your debtor, and am, with true reſpect,

*Philadelphia,* Your obliged friend and humble ſervant,

*January* 29, 1802. GEORGE PFEIFFER.

